# Serious Games as a Method for Enhancing Learning Engagement: Student Perception on Online Higher Education During COVID-19

**DOI:** 10.3389/fpsyg.2022.889975

**Published:** 2022-04-27

**Authors:** Manuel Arias-Calderón, Javiera Castro, Silvina Gayol

**Affiliations:** Departamento de Ciencias Biológicas, Facultad de Ciencias de la Vida, Universidad Andrés Bello, Santiago, Chile

**Keywords:** serious games, gamification, engagement, active learning, education innovation, student motivation

## Abstract

The COVID-19 pandemic has enforced social isolation in many countries worldwide, which forced teachers at all levels of education, including the university context, to adapt new teaching strategies. This study presents a method developed in this regard, that is, serious games were used as a complement to synchronous online classes to ensure the continuity of pedagogical activities in a physiology course at Universidad Andrés Bello, Chile. Using serious games is a strategy in the field of gamification, which is a commonly used learning strategy for online teaching as necessitated by COVID-19. This study is quantitative in nature and conducted a questionnaire survey on 108 second-year undergraduate nursing students to determine their perception about this innovation. The results demonstrate that the students well valued the proposed pedagogical innovative model in terms of motivation and engagement. Moreover, they reported that the model can serve as a meaningful learning experience. These perceptions suggest that the model is an efficient strategy for implementing the physiology curricula in the context of online teaching. Moreover, the results imply that the model should be applied to other courses and disciplines in the undergraduate program and provide support that it is a valid strategy for face-to-face teaching. Lastly, the finding points to the potential of the model to be explored as a learning strategy in the age of education post-COVID-19.

## Introduction

The COVID-19 outbreak disrupted the teaching and learning processes in higher education given the context of social isolation required to mitigate the spread of the pandemic. The ensuing scenario led to the rapid transition to online pedagogical strategies to ensure the continuity of the learning process and to provide implement social distancing at the same time. After learning shifted to online platforms, many areas were more affected than others due to the abrupt transition to online lectures and activities. At the university level, online transition was particularly difficult for courses and undergraduate programs in the STEM fields (science, technology, engineering, and math) and health sciences. The reason underlying this notion is the specific features of learning strategies in these areas, including active lectures, hands-on laboratories, and research projects, among others, which require highly specific technology for developing online courses (Iheduru-Anderson and Foley, 2021; Pagoto et al., 2021; Jeffries et al., 2022). These characteristics of the time of crisis in higher education have also been present in the COVID context of Chilean universities. Some of the most important challenges in our country are related to three major areas: financial, inequality and learning logistics (Funk, 2021). The COVID outbreak has forced the higher education system in Chile, as in the rest of the world, to rapidly develop new didactics and pedagogical strategies to ensure continuity of learning (Funk, 2021; Guzmán Droguett et al., 2021). The training of higher education staff and academics in the use of new technological tools and platforms has been one of the main responses implemented both at governmental and university level, identifying that the use of appropriate technology was essential for the continuity of the teaching-learning processes during the pandemic.

In this context, COVID-19 has forced institutions to shift from traditional face-to-face classes to online ones by initially adopting emergency learning strategies, which are temporary adaptations during the crisis due to the lack of time for planning. Moreover, intentional strategies were later adopted, where a pedagogical team not only delivers contents but also develops strategies centered on learning (Cruea, 2020; Rapanta et al., 2020; UNESCO, 2020). Among others features, online modalities require flexibility and access to technology for students and teachers to ensure the continuity of learning processes. However, important negative issues have emerged from these online strategies, such as loss of student motivation and engagement, decreased attention, poor time management, and negative effects on the psychosocial well-being of students, which result in impaired learning (Rivera-Vargas et al., 2021). Face-to-face teaching, particularly for subjects and programs under the health sciences, is irreplaceable. However, the use of technologies can be incorporated to assist the teaching process during times of crisis. This aspect can be considered an opportunity to develop directed teaching strategies based on technology, which can be used to complement and strengthen learning processes during times without crises (Lira et al., 2020). Thus, the challenge is to develop strategies that encourage student autonomy and to increase motivation, engagement, and attention, which are substantial elements for learning improvement.

Several teaching strategies have been developed to ensure academic success during the pandemic with the objective of maximizing the knowledge acquisition of students by enhancing motivation, self-efficacy, and engagement (Hartnett, 2016; Hsu et al., 2019; Chiu et al., 2021). Among the different strategies, the use of games or game elements in a non-entertainment environment to promote learning is generally known as *gamification* (Dichev and Dicheva, 2017), which has been implemented in online courses. The use of this method has produced positive results in the COVID-19 context (Nieto-Escamez and Roldán-Tapia, 2021; Rincon-Flores and Santos-Guevara, 2021). Moreover, gamification has been used in different subjects, such as business (Pakinee and Puritat, 2021), computer sciences (Liénardy and Donnet, 2020), and chemistry learning (da Silva Júnior et al., 2020a,b), as well as biological sciences courses, such as biology (Kalleny, 2020; Lobet et al., 2021), microbiology (Dustman et al., 2021), physiology (Moro et al., 2020; Hennekes et al., 2021; Herkes et al., 2021; Kane et al., 2022), and medical and nursing education (Gentry et al., 2019; Malicki et al., 2020; O’Connell et al., 2020; van Gaalen et al., 2021).

Against this background, this study presents an experience using gamification, in a format of serious-games activities for learning, in a physiology course for nursing students at Universidad Andrés Bello. The implementation of these activities in online teaching enabled us to ensure the continuity of our learning process with the students during academic confinement due to the COVID-19 pandemic. The results of the analysis provide answers to the following research questions (RQ) in terms of the appraisal of the students about the gamified learning experience:

RQ1: What is the appraisal of students about using serious games for learning experience and engagement?

RQ2: What are the possibilities and limitations of these gamification strategies?

## Theoretical Framework

Developing effective learning through student-centered strategies implemented in online teaching requires that students develop several characteristics, such as self-discipline, motivation, and autonomy (Goulão and Menedez, 2015; Kirmizi, 2015; Roddy et al., 2017; Duchatelet and Donche, 2019). These features are relevant for online learning across all disciplines, including the sciences (DiBenedetto and Bembenutty, 2013; Higgins et al., 2021a,b) and health courses (Hayat et al., 2020; Shorey and Lopez, 2021). In the context of the study, physiology for undergraduate students in the health sciences is a particularly challenging subject, because it presents high levels of complexity. Furthermore, it requires a solid foundation by understanding the basic concepts of the biological sciences, as well as by relating these concepts with those of the subject to understand the functions of the different systems of humans (Michael, 2007; Slominski et al., 2019). It also presents large amounts of content imparted through time-limited lectures, which requires the need for significant engagement, self-efficacy, and autonomous work among students to achieve the desired learning outcomes.

A strategy widely used to improve learning in online education during the COVID-19 pandemic has been gamification, which applies different game formats to the learning environments [see the review by Nieto-Escamez and Roldán-Tapia (2021)]. Using games in various educational contexts has been previously investigated, where positive results were obtained in terms of learning improvement (Dichev and Dicheva, 2017). Recent research demonstrates that using games in education promotes social interaction (Waytz and Gray, 2018), improves mental health (Cruea, 2020), and reduces isolation (Valkenburg and Peter, 2009). These results suggest that using games promotes a state of well-being that facilitates meaningful learning.

Using games in education is a strategy that requires the active participation of students in the learning process (Campillo-Ferrer et al., 2020). To ensure effectivity, games must promote interaction; be creative and engaging; and be a motivator to stimulate participation and deliver immediate feedback (Lira et al., 2020; Nieto-Escamez and Roldán-Tapia, 2021). Several studies demonstrate that games, in general, encourage creativity and innovation; increase engagement, self-efficacy, and motivation; encourage collaboration; improve attention and concentration; and improve academic performance (Banfield and Wilkerson, 2014; Nietfeld, 2018; Campillo-Ferrer et al., 2020; Nieto-Escamez and Roldán-Tapia, 2021).

Among the types of games developed, serious games or games for learning are those that focus on education, information delivery, and practice of skills. In a broader definition, serious games could be understood as an interactive computer application for educational purposes, which has a challenging objective, incorporates some concept of scoring, and imparts in the user a skill, knowledge or attitude that can be applied in the real world (Bergeron, 2006). The design of serious games considers different features such as tracking correct answers as assessment, environment including game fiction, and a challenge proposed with rules to achieved it, with or without a complex hardware design and with different player-game interaction styles, from traditional ones using keyboard and mouse click, to virtual reality modes (Bedwell et al., 2012; Gorbanev et al., 2018). In terms of the educational purpose, the benefits of serious games can be explained from different pedagogical perspectives, including constructivism, humanism and cognitive, facilitating learnings and skills development in different areas from business and industry to medical education (Wu et al., 2012).

Serious games are learner-centered approaches, where the student controls the learning process in an interactive manner (Ricciardi and De Paolis, 2014). For example, educational computer games enable students to elaborate strategies, achieve goals, and consider previous knowledge when facing a problem-solving situation, which is expected to develop cognitive skills, such as memory, attention, and critical thinking (Rondon et al., 2013). However, the results of studies on serious games are non-conclusive when comparing the acquisition of contents and performance in tests between online and traditional learning (Rondon et al., 2013; Nieto-Escamez and Roldán-Tapia, 2021). Nevertheless, students report increased motivation when information is delivered through serious games (Nieto-Escamez and Roldán-Tapia, 2021), including when serious games are applied specifically to physiology courses (Moro et al., 2020). Due to the huge variation in the design of the experience, a comparison among studies is difficult. However, certain key features emerge as critical to learning improvement and motivation, such as clear rules and goals, degree of entertainment, questions of appropriate complexity, feedback, and new problems to be solved (Bellotti et al., 2013; Rondon et al., 2013).

Given the context and theoretical framework, the current study developed a learning model experience using serious games for application to a physiology course for undergraduate nursing students. The general objective of this study is to assess the appraisal of students in terms of whether the application of game-base pedagogy can enhance their behavioral and attitudinal skills for learning through online education during COVID-19 at Universidad Andrés Bello in Chile.

## Materials and Methods

This section presents a description of the teaching innovation model; the context in which it was developed; the methodology used in the research; and the procedure for analyzing the results.

### Innovation Context

The physiology course is offered during the first semester (March–August) of the academic year for undergraduate nursing students in their second year at Universidad Andrés Bello in Santiago, Chile. A total of 267 students, who were enrolled in the physiology course at the first semester for 2021, were invited to participate. The course was conducted entirely in an online format to students without previous university experience in face-to-face lectures due to COVID-19-related lockdown, which occurred during academic year 2020 (the first year of university for students).

The cohort was divided into four class sections for lectures and synchronous activities during the semester and provided with access to a *virtual classroom* supported by a learning management system (LMS) called Blackboard.

Human physiology courses for health careers present different curricular approaches in institutions around the world, ranging from an integrated model that teaches aspects of physiology in conjunction with other basic biological sciences to the more traditional model of teaching physiology in a systems-based, non-integrative nature with other areas of biological knowledge. Both pedagogical approaches present benefits and challenges in their implementation (Hasan and Sequeira, 2012; Fernando et al., 2020; Adams and Dewsbury, 2022), and both systems are currently considered valid for the teaching of human physiology. In our case, at Universidad Andrés Bello in Chile, the teaching of human physiology for nursing students is based on a curriculum whose didactic approach is based on systems-based teaching. The syllabus is classified into seven units, where each is associated with the specific concepts and mechanism of one system of the human body. All units are evaluated using a summative final assessment with multiple-choice questions. Among the conceptual units of the course, our innovation was exclusively developed in the context of endocrine physiology, which is particularly difficult unit among those defined by our syllabus, because the subject requires a solid theoretical foundation on previous courses in basic sciences.

### Pedagogical Model Design

The objective of the study was to generate an educational strategy model that enables students to learn in a playful environment and allowing them to select where and when they can conduct the activities. Moreover, instant feedback is ensured during this process. At the same time, we aimed to motivate students to take responsibility for their learning and to collaborate in organizing their study time.

We developed five serious game activities^1^ using the Genially© platform. The design of the games was based on templates and extensions suggested by the platform (e.g., escape room and quizzes). The games progressed through increasing levels of difficulty and comprised content aligned with lower taxonomic levels (memorize or recall) to higher abilities (advanced relations and application). The final game (number five) was proposed as an integrative activity, which is based on a clinical case study that includes all topics covered by the lectures. The objective of the first four games was to consolidate the concepts discussed in each online synchronous lecture and to self-assess learning in the intervened unit using the previous game activity. The games contain different teaching and assessments activities, such as drag-and-drop classification, paired-item questions, conceptual maps, and multiple-choice questions. Each activity includes feedback for answers and access to complementary learning resources. All games were proposed as asynchronous activities to the students.

From a didactic perspective, the games were design in terms of brief interactive activities which strengthen the content previously delivered by synchronous lecture, based in a flipped classroom methodology. The learning objective for each game is aligned with one of the learning objectives assessed in this unit of the syllabus. For example, in Game 1, students are faced to a breakout room game type, related with general characteristics of hormones and its mechanism of action. Through three different “missions,” in a clicker mode of interaction, students answer questions about hormone classification, solubility properties, types of receptors, and molecular mechanisms. The learning objective for this game was classify hormones by its chemical nature and recognize action mechanism related with receptor type. In games 2, 3, and 4, a multiple-choice questions game was designed. In this games, different hormonal axis and its effects were assessed, in which student complete missions by selecting the correct answers. For these games, learning objectives were recognize the hormonal axis and its regulation mechanisms, and relates hormones to their physiological effects. For the final game (Game 5) a context of medical emergency was given to the game, which was design based on clinical cases. Students resolved the case by answered multiple-choice questions, with a learning objective of relates hormones alterations with several sign and symptoms described in the patient of the clinical case. For the final multiple choice question assessment of the unit, the same learning objectives were evaluated for all students, including those who voluntary decided not to play games.

The games were delivered sequentially to the students immediately after a corresponding synchronous lecture (one lecture per week) across 6 weeks. Games 1 and 2 were released at week 1; Game 3 during week 2; and Game 4 during week 3. Lastly, Game 5 (the integrative concepts game) was released during week 6 given its connotation of a gamified activity for the preparation of the final evaluation of the unit. The students accessed the games through individual accounts on the LMS (Blackboard). Doing so enabled us to monitor student access and usage data per game. The general instructions for these activities were presented to the students in the first synchronous lecture of the topic. They were informed that playing games is an individual choice they make. Given this context, the game was available online to students 24/7 with no limited attempts per student for each game.

Participation in the study was considered voluntary, where the students also provided informed consent. Participation was tracked according to the number of game access through LMS. The frequency of game access exerted no influence in the assessment grade for multiple- and non-playing students.

### Research Methodology

To analyze the appraisals of the students about their learning experience using serious games, the study developed a quantitative approach based on data obtained from the survey.

#### Instrument

The appraisal of the students was assessed through the online survey, which was designed by the authors using Microsoft Forms and was associated with institutional e-mail accounts of the students. The survey comprised 15 items rated using a five-point Likert-type scale (Joshi et al., 2015) and assessed the extent to which the students “strongly agree” to “strongly disagree” (from 5 to 1 at Likert-type scale) to the statements. The survey was disseminated at the end of the examination period of the unit (after the final summative assessment) over a 2-week period. The reliability of the test was assessed using Cronbach’s alpha (α). The survey design also contains a final open-ended question, where students were invited to submit any positive or negative perception regarding any aspect of the activities related to serious games.

#### Sampling Design

The participants (*n* = 267) were invited to use the serious game activities designed as a complementary material for synchronous lectures. Out of the total participants, 211 (79.03%) conducted one of the games at least once during 6-week endocrine physiology unit. A total of 3,358 games were played with attempts ranging from 1 to more than 50 times and with Game 1 as the most played one. During the intervention, the students conducted the activities during all days of the week with high rates of access from Wednesday to Friday, whereas usage was preferent at night hours.

Out of the 267 students, the study obtained 108 valid responses to the survey for a response rate of 40.5%. Cronbach’s α (test reliability) reached 0.92 (high reliability). The error for the sample was set to 7% with a confidence level of 95%. Unfortunately, the pandemic situation, which forced the transition to online classes, did not enable sufficient interaction with students to motivate them to answer the survey. In addition, the survey was available to students at the end of the semester, which is the time of the year in which students are overloaded with activities at the end of the academic semester.

Moreover, evidence exists that online surveys are much less likely to achieve response rates as high as those administered on paper despite the use of various practices to increase this rate. Data clearly indicate that the face-to-face administration of surveys results in high response rates (Nulty, 2008; Yetter and Capaccioli, 2010). In addition, Silva and Duarte (2014) argued that most individuals use the Internet for entertainment and recreation, which makes them discount requests for participation in research surveys, which results in a low response rate. These factors should be considered when determining the margin of error.

#### Analysis Proposal

Data obtained from the survey were recorded and analyzed anonymously. The results of the Likert-type rating are presented as a percentage of responses (or the total number of responses) for each question. For description and interpretation, the percentages for *strongly agree* and *agree* were considered positive results; *neither agree nor disagree* was considered neutral; and *disagree* and *strongly disagree* were noted as negative results.

For analysis, data were classified into four categories, which group the questions related to the competencies and skills considered important to online learning, as described in the theoretical framework (Goulão and Menedez, 2015; Kirmizi, 2015; Roddy et al., 2017; Duchatelet and Donche, 2019; Ferrer et al., 2022). The four categories are attitudinal competencies (three questions), student self-efficacy (three questions), academic performance (six questions), and the student’s perception of future impact (three questions).

The category of attitudinal competencies enables the analysis of the results in relation to the manner in which the method used enhances the motivation and engagement of students in learning. Student self-efficacy is related to aspects associated with self-discipline, autonomy, and time management and their impact on student learning. Aspects related to academic performance are analyzed in terms of the evaluation of the appraisal of the students about the usefulness of the strategy using serious games for the preparation of summative course assessments. Finally, analysis considers the perception of the students of the future impact of the method, in which responses are analyzed in relation to their assessment of the usefulness of this strategy in future learning scenarios, such as face-to-face learning activities, other units of the physiology course, and its use in other subjects under the nursing undergraduate program.

For questions with high neutral or negative results, the opinions of the students as elicited by the open-ended question in the survey are presented as examples of detailed information for exploratory analysis. Excerpts of the opinions of students are presented as quotes in the section “Results.”

## Results

The results of the survey were presented as the percentages of responses for each question, which were classified according to the abovementioned categories. A general analysis of the results indicates high frequencies of the responses *strongly agree* or *agree* for the majority of the questions. This finding suggests that the students generally positively evaluated the use of serious games as a learning strategy.

### Attitudinal Competencies

Questions on the motivation, engagement, and confidence in concepts of the students were classified under this category (Table 1). In terms of motivation, 87.9% of the respondents report that performing serious game activities improves motivation to study the theoretical contents associated with the activities (strongly agree: 54.6%; agree: 33.3%). Moreover, 90.8% (strongly agree: 63%; agree: 27.8%) perceived that performing the serious games improved their engagement in the learning process. In relation to confidence in the concepts learned, 86.1% reported that this strategy improved their confidence. These results align closely to one of our objectives of the use of serious games as learning strategy, which suggests its effectiveness in enhancing motivation, engagement, and confidence in learning.

**TABLE 1:** Percentage of responses to questions on attitudinal competencies.

Questions	Strongly agree	Agree	Neither agree nor disagree	Disagree	Strongly disagree
Playing the serious games increased your motivation to study	54.6% (59)	33.3% (36)	8.3% (9)	3.7% (4)	0.0% (0)
Serious games improve your engagement in the learning process	63.0% (68)	27.8% (30)	8.3% (9)	0.9% (1)	0.0% (0)
Performing serious games improve your confidence in concepts learned	60.2% (65)	25.9% (28)	7.4% (8)	5.6% (6)	0.9% (1)

### Self-Efficacy

Concepts related to this category include the perceptions of students about their abilities and behaviors to learn and to accomplish their academic activities. In this line, we analyzed the appraisal of the students regarding improvement and attention and concentration the optimization of time management to examine physiology concepts using serious games as learning activities.

Table 2 presents the results, where more than 70% of the respondents believe that playing serious games improved concentration during study, whereas more than 80% (totally agree: 55.6%; agree: 26.9%) reported that playing serious games optimized their personal time for study. Moreover, 74.1% of the students agree (or strongly agree) with the fact that serious games improve concentration and attention during synchronous classes. The results strongly suggest that using serious games as a learning strategy exerted a positive effect on the self-efficacy of students, which is relevant for meaningful learning.

**TABLE 2:** Percentage of responses to questions on self-efficacy.

Questions	Strongly agree	Agree	Neither agree nor disagree	Disagree	Strongly disagree
Playing the serious games improves your concentration during self-study	44.4% (48)	33.3% (36)	18.5% (20)	2.8% (3)	0.9% (1)
Performing serious game activities optimize personal study time	55.6% (60)	26.9% (29)	13.9% (15)	1.9% (2)	1.9% (2)
Your concentration and class attention improves by performing serious game activities	42.6% (46)	31.5% (34)	19.4% (21)	2.8% (3)	3.7% (4)

### Academic Performance

The questions in this category (Table 3) are intended to evaluate the assessment of the students in terms of whether using serious games directly prepared them to obtain better outcomes in the summative assessments of the unit after the application of the strategy.

**TABLE 3:** Percentage of responses to questions on academic performance.

Questions	Strongly agree	Agree	Neither agree nor disagree	Disagree	Strongly disagree
Playing the serious games improves the learning of the associated concepts	59.3% (64)	31.5% (34)	8.3% (9)	0.9% (1)	0.0% (0)
Performing serious games facilitates doing seminar activities	36.1% (39)	24.1% (26)	27.8% (30)	11.1% (12)	0.9% (1)
Performing serious games facilitates study for weekly assessments	32.4% (35)	25.0% (27)	29.6% (32)	11.1% (12)	1.9% (2)
Performing serious games facilitates study for final assessment	40.7% (4)	32.4% (35)	13.9% (15)	7.4% (8)	5.6% (6)
Performing serious game activities was a significant learning experience	60.2% (65)	25.0% (27)	13.9% (15)	0.0% (0)	0.9% (1)
Do you feel well prepared for unit assessment by performing serious games	44.4% (48)	29.6% (32)	14.8% (16)	8.3% (9)	2.8% (3)

The results indicate that 90.8% of the students considered that serious games improved learning, whereas 85.2% positively appraised that playing serious games was a meaningful learning experience.

Among the survey questions, a high frequency of neutral responses (neither agree nor disagree) was obtained for questions on whether serious games facilitated the preparation of the students for the weekly summative assessments of the subject (57.4%). The same result was found for the question on whether serious games are useful for the preparation of the seminar activities of the course, which are problem-solving or case-study activities conducted on a weekly basis (29.7%). Both questions also exhibited the highest negative percentage in the survey at 13 and 12%, respectively. Furthermore, only 44.4% of the respondents considered the performance of serious games to be positive in the preparation for the final assessments of the unit (neutral responses: 14.8%; negative responses: 11.1%).

Considering these questions with high neutral and negative results, we conducted an exploratory analysis of the opinions of the students using responses to the open-ended question to correlate the results with specific information. Several opinions from students are in line with the neutral/negative appraisal of the relationship between serious games and summative assessments. This aspect provided certain clues about the lack of alignment between game content and the level of difficulty of assessments. The following excerpts are examples of the opinions of the students correlated with this observation.

*The questions in the gamified activities should have been more application-oriented because most of the questions were memory-oriented, however, the activity is quite innovative and engaging. Maybe the games should be more similar to the assessments in difficulty, with a limited time to answer, with better feedback for the wrong answers*…. *I think that if the activities were a little more complex, with more final assessment style questions, they would be more useful, although they are already good*… *The activities should be more difficult, because they are based on basic topics, and there is no equivalency in the assessments of the course.*

These opinions are consistent with the perception that the contents of the serious games are only partially aligned to the difficulty levels of assessments, which suggests a possible explanation for the high rates of the neutral/negative results related to academic performance appraisals and the preparation of students for assessments using serious game activities. Nevertheless, the results indicate that 90.8% of the students positively appraised the use of serious game for improving their learning about the concepts of the unit.

### Future Impact

To evaluate the perceptions of the students whether serious games should be applied to other learning contexts, such as in face-to-face teaching or in other courses of their undergraduate program, three questions were analyzed. Table 4 presents the results, which indicates that 81.7% of the students considered that the application of serious games to other courses could exert a positive impact. In addition, more than 90% of positive responses were obtained when asked about their perception of whether these activities will be useful in the face-to-face format of the course and whether using serious games in the entire physiology course would improve academic performance.

**TABLE 4:** Percentage of responses to questions on the perception of students about the future impact of the model.

Questions	Strongly agree	Agree	Neither agree nor disagree	Disagree	Strongly disagree
Other lectures would be more significant if they used serious game activities	67.6% (73)	24.1% (26)	6.5% (7)	0.9% (1)	0.9% (1)
The serious game activities will be useful in a face-to-face lecture mode	80.6% (87)	13.0% (14)	3.7% (4)	1.9% (2)	0.9% (1)
Using serious game activities in all units of the lecture will improve students’ performance	80.6% (87)	13.9% (15)	4.6% (5)	0.0% (0)	0.9% (1)

These results of the four categories suggest that the application of serious games should be considered as a positive strategy for future courses, including physiology, in face-to-face learning.

## Discussion

This article presented the result of our evaluation of the appraisal of students regarding the use of serious games as a learning strategy for online learning, which was imposed in Universidad Andrés Bello, Chile, due to the COVID-19 outbreak. Four main categories emerged from the survey on student perception with the objective of characterizing the proposed pedagogical model in an exploratory manner. In general, the students well received the model given its nature as a method for enhancing learning. However, certain results are important to the discussion of previous research on gamification.

In the current study, the majority of students reported improvements in motivation, confidence, and engagement to study the theoretical concepts of the subject using serious games. Gamification has been related to increased intrinsic and extrinsic motivation in learners (Rutledge et al., 2018; Moro et al., 2020; Nieto-Escamez and Roldán-Tapia, 2021). Moreover, goal setting and flow theories link enhanced motivation and engagement through gamification activities given that the learners present an appropriate state of well-being (Landers, 2014; Huang and Hew, 2018). Considering the results for attitudinal competencies and the general perception that physiology is a difficult subject (Michael, 2007; Slominski et al., 2019), the use of serious games may be associated with a positive effect in the well-being of students, which could be related to their perceptions about motivation, engagement, confidence, and overall learning enhancement appraisal, according to theories. This aspect is important to online teaching given the negative psychological effects of COVID-19-related lockdown in students. Nevertheless, further research and appropriate assessment of the psychological impacts of the use of serious games in the context of online learning is required.

Furthermore, the results demonstrate a highly positive appraisal in enhancing self-discipline, autonomy, and personal time management for study among the students. This finding is related to results for self-efficacy. Along this line, the results are aligned with those of previous research, which demonstrated that the use of games in the learning context is associated with statements related to self-determination theory (Landers, 2014; Richter et al., 2015). Scholars propose that enhancing autonomy and other self-efficacy features in students is an important characteristic for effective learning, particularly in online environments (Goulão and Menedez, 2015; Roddy et al., 2017; Duchatelet and Donche, 2019). Moreover, the results are in line with those of previous research that reported that gamification promotes the autonomy of students as an important factor for positives outcomes when using games as a learning strategy (Banfield and Wilkerson, 2014; Landers, 2014; Campillo-Ferrer et al., 2020; Rincon-Flores and Santos-Guevara, 2021).

In the context of using gamification strategies for online teaching during COVID-19, several studies report enhanced learning outcomes based on questionnaires and objective test results that focus on the perception of students (Nieto-Escamez and Roldán-Tapia, 2021). In the current study, the appraisals of the students were more negative in terms of being well prepared for objective assessments. The preliminary exploration of the opinions of students in relation to academic performance suggests a probable misalignment between the design of the content of serious games and the levels of difficulty of assessments. These results emphasized that a review of the design of our game elements and disciplinary content is necessary to improve learning outcomes using the proposed pedagogical model. This notion is presented given that clear rules and goals in the game, an appropriate degree of entertainment, questions with appropriate complexity, and feedback are key features for improving learning through gamification (Bellotti et al., 2013; Rondon et al., 2013). An assessment of objective test scores is also required for the appropriate validation of the proposed pedagogical model in terms of performance in academic test. Apart from the high frequencies of neutral appraisal by the students about preparation for assessments using serious games, the results highlight that the students considered the use of serious games in physiology a meaningful learning experience.

The study presented a pedagogical model, which uses serious games as a complement to synchronous lecture in online teaching. The model comprises five game activities, which are in line with gamification characteristics. It intends to support the teaching of a particular unit of the physiology course, which students defined as a very difficult subject in the undergraduate nursing program. Based on the perceptions of students, the students well valued the pedagogical model in terms of enhancing motivation and engagement to learn. The results, which are in line with those of several studies on the use of gamification in undergraduate biological sciences and physiology courses, enabled the validation of the model for application to the learning context, especially online. Moreover, the results demonstrate that the students highly appraised the implementation of the pedagogical model to the rest of the physiology syllabus and to other subjects in the undergraduate program. This finding suggests the possibility of continuing the use of this model not only for the physiology course but also for other courses in biological sciences and other disciplines. Previous studies on gamification have previously described this aspect (Dichev and Dicheva, 2017; Gentry et al., 2019; Malicki et al., 2020; Moro et al., 2020; Dustman et al., 2021; Nieto-Escamez and Roldán-Tapia, 2021; Pakinee and Puritat, 2021; Rincon-Flores and Santos-Guevara, 2021; van Gaalen et al., 2021).

This pedagogical model, which generates a playful learning environment, can serve as an innovation strategy for online or hybrid education systems, because this more frequent environment for students promotes learning in a more relaxed environment, reduces anxiety, and avoids a stressful or overwhelming environment (Iheduru-Anderson and Foley, 2021). On the one hand, previous studies reveal that students prefer hybrid education systems, multiple resources, and complementary technologies (Pagoto et al., 2021). On the other hand, society must be prepared for the threat of a new pandemic, which may replicate the teaching conditions experienced in 2020 and 2021. Along this argument, the current results point to a highly valued relationship between the perceptions of students and the use of this model of learning through serious games in face-to-face learning, which is one of the challenges for the field of education in the post-COVID-19 era. Nevertheless, we suggest that further research is required to evaluate the other benefits of using serious games to achieve desired learning outcomes. This need emerges due to the lack of evidence to date that support gamification or serious games as better strategies than traditional ones in terms of outcomes. Further research is also required to analyze the psychology and theories that underlie the positive results presented in this paper in particular and on the use of serious games in general, and about the transition from COVID-online teaching back to face-to-face teaching.

## Conclusion

The study concludes that the proposed pedagogical model is highly appraised by students in terms of enhanced motivation, engagement, and meaningful learning experience. This educational strategy model seemingly improved learning in a specific area, as we applied it to a physiology course. However, the method can improve transversal cognitive skills that contribute to the development of the students. In the last few years, the advance of social networks, online games, and various technological tools have prompted teachers to incorporate them into formal learning activities.

The COVID-19 outbreak of the last few years has *hastened* this decision not only due to the impossibility of conducting face-to-face classes but also due to objective or maintaining the attention of students during online classes.

Within this framework, integrating serious games to the learning of concepts that may be complex for students is a seemingly attractive idea. In our case, the application of serious games to the learning of the concepts of one of the units of the curriculum was very well received and valued by the students. In turn, the students provided the researchers with opinions and recommendations for improving these activities. The pedagogical model is a type of a strategy based on learning and serves as an environment that students can use at any moment deemed pertinent. This flexibility enables the students to self-manage learning and self-evaluate the lesson contents. However, further research is required to analyze the effect of this model on objective assessments scores and other psychological characteristics related to the learning of complex subjects, such as physiology.

Based on these results, we provided responses to the research questions as follows:

### RQ1: What Is the Appraisal of Students About Using Serious Games for Learning Experience and Engagement?

Students perceive that performing serious game activities improved not only their motivation and commitment to the learning process but also their confidence. Although they do not perceive that playing serious games facilitated their preparation for the weekly summative tests and the final evaluation, they believe that it improved concentration during study and optimized personal study time. In addition, they indicate that serious games enhanced learning and considered them meaningful learning experiences. At the same time, they indicate that performing serious games could exert a significant impact if applied to other subjects; that their use would be useful in face-to-face learning; and that using serious games throughout the subject would improve academic performance. The data are supported by the opinions expressed by the students in the open-ended question.

The students perceive the physiology course as an especially difficult discipline in the field of health. A few of the factors associated with this perception are the nature of the discipline, the traditional teaching methodology (mainly through lectures, where an extensive amount of content is covered within limited periods of time), and the requirement of higher-order cognitive skills to which the students are unaccustomed to using (Michael, 2007). In this context, a change in the traditional didactics of teaching can be considered a good strategy especially through the incorporation of methodologies that enable students to approach concepts considered complex in an attractive manner and related to the technological tools they frequently use. Given the perception of the students about the physiology course, serious games can provide an interesting environment for knowledge acquisition, improvement in interest, motivation, and the enhancement of the learning achievements of the students.

### RQ2: What Are the Possibilities and Limitations of These Gamification Strategies?

Regarding the limitations and given the perceptions of the students, certain contents of serious games should be reviewed and modified, such that they fit better with the different evaluation formats used in the course. However, it is not only necessary to analyze the coherence of the serious games with the strategies and cognitive levels of the subject assessments, but it is also relevant to analyze our pedagogical proposal of the use of serious games in terms of the pedagogical-didactic design of the whole teaching process. It is possible that the most negative perceptions obtained in this study, related to the students’ opinions about playing serious games and the perception of readiness for the subject assessments, is due to the fact that in the didactic sequence in which our serious games are used, the pedagogical objective with which it was designed is lost, and that finally the students understand the activity from a viewpoint of preparation for the evaluation, rather than as a learning activity in itself. Further didactic design research is required to add new evidence to the use of serious games in different didactic sequences for physiology and other basic sciences subjects.

This revision, which is based on feedback obtained from surveys through the comments of students, will enable us to optimize the game contents and design to enhance the perception of students regarding the use of serious games to improve their preparation for summative assessments. Further research should consider other sources of information, such as objective test results (for a quantitative analysis of the effectiveness of serious games), and/or interviews and discourses analyze from students and teachers (regarding the contribution to and support of the model to the overall learning process), to strengthen the pedagogical and psychological conclusions about using serious games as an didactic strategy for enhance learning of physiology and others biological sciences areas.

In terms of possibilities, we believe that these activities cannot only be improved but can also be useful for application to other units of the course and other disciplines beyond physiology or biological sciences courses. The evaluation of the students suggested that the model can be used in other units of the same course and even in other subjects of the same undergraduate program. Considering that our curricular approach to the subject is systems-based, this type of strategy could be useful for a didactic transition toward a more integrated study of the function and regulation of human physiology. In addition, this type of strategy can be used in other basic science subjects outside the area of physiology.

In recent times, scholars have questioned the forms of assessment used in the academic environment. Thus, serious games could be used as an alternative method for assessing student learning. In fact, serious games can be used to revalidate concepts discussed in previous classes to reinforce learning, which presents increased difficulty for students. The model can also be used as an evaluative method for synchronous and asynchronous activities. Therefore, they can be used for activities related to online and face-to-face learning. Based on the results, which indicated that more than 90% of the students reported that serious games can be useful for face-to-face learning, we suggest the use of serious games as an innovative strategy during face-to-face learning in the post-COVID-19 era.

Our results presented here are in line with several previous reports about using gamification and serious games, and its positive effects on learning, but we encourage to keep assessing this methodology in different disciplines and courses, particularly in the context of return to face-to-face teaching mode around the world, exploring the effects of gamification in the post-COVID age.

## Data Availability Statement

The raw data supporting the conclusions of this article will be made available by the authors, without undue reservation.

## Ethics Statement

Ethical review and approval was not required for the study on human participants in accordance with the Local Legislation and Institutional Requirements. The patients/participants provided their written informed consent to participate in this study.

## Author Contributions

SG and MA-C conceived and designed the research. SG, MA-C, and JC developed the games activities, analyzed the data, interpreted the results, and drafted, edited, and revised the manuscript. All authors approved the final version of the manuscript.

## Conflict of Interest

The authors declare that the research was conducted in the absence of any commercial or financial relationships that could be construed as a potential conflict of interest.

## Publisher’s Note

All claims expressed in this article are solely those of the authors and do not necessarily represent those of their affiliated organizations, or those of the publisher, the editors and the reviewers. Any product that may be evaluated in this article, or claim that may be made by its manufacturer, is not guaranteed or endorsed by the publisher.

## Footnotes

1 All serious games developed are free to access (in their original language – Spanish) through following links: Game 1: https://bit.ly/Game1-UNAB; Game 2: https://bit.ly/Game2-UNAB; Game 3: https://bit.ly/Game3-UNAB; Game 4: https://bit.ly/Game4-UNAB; and Game 5: https://bit.ly/Game5-UNAB.
